# Sustained human outbreak of a new MPXV clade I lineage in eastern Democratic Republic of the Congo

**DOI:** 10.1038/s41591-024-03130-3

**Published:** 2024-06-13

**Authors:** Emmanuel Hasivirwe Vakaniaki, Cris Kacita, Eddy Kinganda-Lusamaki, Áine O’Toole, Tony Wawina-Bokalanga, Daniel Mukadi-Bamuleka, Adrienne Amuri-Aziza, Nadine Malyamungu-Bubala, Franklin Mweshi-Kumbana, Léandre Mutimbwa-Mambo, Freddy Belesi-Siangoli, Yves Mujula, Edyth Parker, Pauline-Chloé Muswamba-Kayembe, Sabin S. Nundu, Robert S. Lushima, Jean-Claude Makangara-Cigolo, Noella Mulopo-Mukanya, Elisabeth Pukuta-Simbu, Prince Akil-Bandali, Hugo Kavunga, Ombotimbe Abdramane, Isabel Brosius, Eugene Bangwen, Koen Vercauteren, Nadia A. Sam-Agudu, Edward J. Mills, Olivier Tshiani-Mbaya, Nicole A. Hoff, Anne W. Rimoin, Lisa E. Hensley, Jason Kindrachuk, Cheryl Baxter, Tulio de Oliveira, Ahidjo Ayouba, Martine Peeters, Eric Delaporte, Steve Ahuka-Mundeke, Emma L. Mohr, Nancy J. Sullivan, Jean-Jacques Muyembe-Tamfum, Jean B. Nachega, Andrew Rambaut, Laurens Liesenborghs, Placide Mbala-Kingebeni

**Affiliations:** 1 Institut National de Recherche Biomédicale, Kinshasa, Democratic Republic of the Congo; 2 Department of Clinical Sciences, Institute of Tropical Medicine, Antwerp, Belgium; 3 Hemorrhagic Fever and Monkeypox Program, Ministry of Health, Kinshasa, Democratic Republic of the Congo; 4 Service de Microbiologie, Département de Biologie Médicale, Cliniques Universitaires de Kinshasa, Université de Kinshasa, Kinshasa, Democratic Republic of the Congo; 5 TransVIHMI (Recherches Translationnelles sur le VIH et les Maladies Infectieuses endémiques et émergentes), Université de Montpellier, French National Research Institute for Sustainable Development (IRD), INSERM, Montpellier, France; 6 Institute of Ecology and Evolution, University of Edinburgh, Edinburgh, UK; 7 Rodolphe Merieux INRB-Goma Laboratory, Goma, Democratic Republic of the Congo; 8 Kamituga General Hospital, South Kivu, Democratic Republic of the Congo; 9 Kamituga Health Zone, South Kivu, Democratic Republic of the Congo; 10 Provincial Health Division, South Kivu, Democratic Republic of the Congo; 11 African Center of Excellence for Genomics of Infectious Diseases, Redeemer’s University, Ede, Nigeria; 12 Medical Department, The Alliance for International Medical Action, Goma, Democratic Republic of the Congo; 13 Department of Pediatrics and Child Health, School of Medical Sciences, University of Cape Coast, Cape Coast, Ghana; 14 International Research Center of Excellence, Institute of Human Virology Nigeria, Abuja, Nigeria; 15 Global Pediatrics Program and Division of Infectious Diseases, Department of Pediatrics, University of Minnesota Medical School, Minneapolis, MN USA; 16 Department of Health Research Methods, Evidence and Impact, Faculty of Health Sciences, McMaster University, Hamilton, Ontario Canada; 17 Frederick National Laboratory, Leidos Biomedical Research, Clinical Monitoring Research Program Directorate, Frederick, MD USA; 18 Department of Epidemiology, Jonathan and Karin Fielding School of Public Health, University of California, Los Angeles, CA USA; 19 US Department of Agriculture, Manhattan, KS USA; 20 University of Manitoba, Winnipeg, Manitoba Canada; 21 Centre for Epidemic Response and Innovation, Stellenbosch University, Cape Town, South Africa; 22 Department of Pediatrics, Division of Infectious Diseases, University of Wisconsin-Madison, Madison, WI USA; 23 National Emerging Infectious Diseases Laboratory, Boston University, Boston, MA USA; 24 Department of Epidemiology, Infectious Diseases and Microbiology, University of Pittsburgh, School of Public Health, Pittsburgh, PA USA; 25 Department of Epidemiology and International Health, Johns Hopkins Bloomberg School of Public Health, Baltimore, MD USA; 26 Department of Medicine, Division of Infectious Diseases, Stellenbosch University Faculty of Medicine and Health Sciences, Cape Town, South Africa; 27 Department of Microbiology, Immunology and Transplantation, KU Leuven, Leuven, Belgium

**Keywords:** DNA methylation, Viral infection

## Abstract

Outbreaks of monkeypox (mpox) have historically resulted from zoonotic spillover of clade I monkeypox virus (MPXV) in Central Africa and clade II MPXV in West Africa. In 2022, subclade IIb caused a global epidemic linked to transmission through sexual contact. Here we describe the epidemiological and genomic features of an mpox outbreak in a mining region in eastern Democratic Republic of the Congo, caused by clade I MPXV. Surveillance data collected between September 2023 and January 2024 identified 241 suspected cases. Genomic analysis demonstrates a distinct clade I lineage divergent from previously circulating strains in the Democratic Republic of the Congo. Of the 108 polymerase chain reaction-confirmed mpox cases, the median age of individuals was 22 years, 51.9% were female and 29% were sex workers, suggesting a potential role for sexual transmission. The predominance of APOBEC3-type mutations and the estimated emergence time around mid-September 2023 imply recent sustained human-to-human transmission.

## Abstract (web summary)

Genomic and epidemiologic analyses of monkeypox cases stemming from a recent outbreak in the Democratic Republic of the Congo suggest that a new clade I lineage is circulating and that its spread may be linked to sexual transmission.

## Main

Monkeypox (mpox) attracted global attention in 2022 due to a widespread epidemic across nonendemic regions with transmission linked to sexual contact. For more than 50 years, mpox virus (MPXV) caused outbreaks in several endemic African countries, predominantly from zoonotic spillover with secondary transmission^1^. MPXV is an enveloped, double‐stranded DNA virus of the *Poxviridae* family, which includes variola (the causative agent of smallpox, now eradicated), vaccinia (used in smallpox vaccination) and cowpox viruses. In humans, MPXV causes mpox, characterized by fever, lymphadenopathy and vesiculopapular rash. Two distinct genetic MPXV clades exist: clade I mainly in Central Africa, especially the Democratic Republic of the Congo (DRC), and is associated with severe clinical symptoms and substantial mortality (4–11%)^2^, whereas clade II, largely confined to West Africa until the 2022 global epidemic, causes less severe illness and lower mortality of <4% (ref. ^2^).

Historically, clade I MPXV has predominated, accounting for 95% of reported cases^1^. In 2017, a major outbreak of clade IIb MPXV occurred in Nigeria, with sustained human-to-human transmission, including through sexual contact^3^. These findings were overlooked until a clade IIb lineage—B.1 (ref. ^4^)—spawned a global epidemic in May 2022, with 95,226 confirmed cases in 117 countries as of March 2024 (ref. ^4^). Genomic analyses of B.1 revealed a mutational pattern suggesting noncanonical evolution, driven by an apolipoprotein B messenger RNA editing enzyme, catalytic subunit 3 (APOBEC3) cytosine deamination, a characteristic feature of MPXV human-to-human transmission^5,6^. MPXV mutations have been confirmed in vitro as stemming from the apolipoprotein B mRNA editing enzyme, catalytic polypeptide-like 3F (APOBEC3F)^7^, suggesting that clade IIb entered human populations as early as 2015 (refs. ^6,7^). As of April 2024, the global clade IIb B.1 epidemic has largely subsided, although the virus continues to circulate in Nigeria and other countries^4^.

As clade II outbreaks have waned, clade I MPXV infections in Central Africa have been increasing^4^, especially in remote forest areas, probably from to zoonotic spillover with secondary human-to-human transmission within households^8,9^. Progressive annual increases in mpox cases were reported in the DRC, with a record 14,626 cases in 2023, possibly indicating a shift toward increased human-to-human transmission^10^. We recently documented a cluster of MPXV infections linked to sexual contact in the DRC^11^. Concurrently, new mpox cases have continued to emerge in several previously unaffected areas of the DRC. In September 2023, the first-ever mpox cases were detected in Kamituga Health Zone, a densely populated mining area in South Kivu Province in eastern DRC. Initial sequencing of six cases from January 2024 by Masirika et al.^12,13^ revealed the presence of a divergent lineage of clade I. In this Brief Communication, we describe the results of an investigation into this outbreak, including detailed genomic analysis of cases dating back to September 2023, to elucidate the origins and nature of this event.

**Fig. 1:**
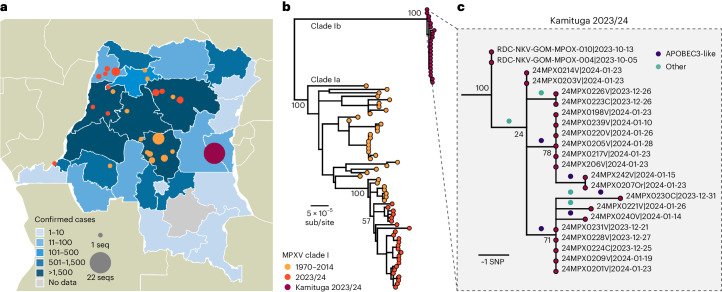
Mapping number of reported mpox cases and genomics analysis, Kamituga, DRC. **a**, Map of the DRC with provinces colored by the number of reported cases. MPXV genomes available are indicated by colored circles (31 January 2024). These are placed at the centroid of the town, the health zone or the province depending on the precision of the recorded location. **b**, Maximum likelihood phylogeny of clade I genomes, including those from the present study (indicated as 2023/24 and Kamituga samples), with all previously sequenced genomes publicly available on GenBank. A version of this phylogeny where individual tips are labeled with accession numbers, locations and dates can be found in Supplementary Figs. 1 and 2. **c**, The Kamituga cluster with reconstructed mutations indicated on the branches. Mutations are colored by whether they are consistent with APOBEC3 deamination (dark blue) or not (green). sub/site, substitutions per site.

Between 29 September 2023, and 29 February 2024, South Kivu’s provincial surveillance authorities recorded 241 suspected cases that met the national case definition for mpox (Fig. 1a). Most cases (93%) were in Kamituga Health Zone. Laboratory specimens from 119 (48.9%) of the patients hospitalized for mpox were collected for polymerase chain reaction (PCR) testing. Of these, 108 (90.8%) were confirmed MPXV positive. Demographic characteristics and clinical manifestations were similar between suspected and confirmed cases (Supplementary Table 1).

Among individuals with PCR-confirmed mpox, the majority were female (56/108, 51.9%) and the median age was 22 years (interquartile range 18–27) (Fig. 1b). Children <15 years constituted 14.8% (16/108) of confirmed cases, individuals aged 15–30 years accounted for 67% (73/108), and 17.6% (19/108) of cases were individuals 30–49 years. Additionally, 28.7% (31/108) of the confirmed cases and 29.5% (71/241) of all suspected cases indicated sex work as their profession during the survey conducted by the provisional health authorities (Methods). None among individuals with confirmed mpox had been vaccinated against smallpox, which was eliminated in the DRC in 1971 with vaccination campaigns ending in 1980 (ref. ^14^).

The DRC Ministry of Public Health’s investigation form for suspected mpox cases included a limited number of clinical variables. All confirmed cases had a cutaneous rash, 64/108 (59%) had fever and 45/108 (42%) had lymphadenopathy. Most suspected mpox cases (91%) were hospitalized mainly for isolation, not for disease severity. Among 108 confirmed cases, 10 (9.3%) were bedridden. We reviewed hospital records for 134 of 241 suspected cases to obtain additional clinical information. Human immunodeficiency virus status was known for 34.3% (46/134), with 6.5% (3/46) positive. Eighty-five percent (114/134 patients) had genital lesions. Two patients with mpox (1.4%) died during hospitalization.

**Fig. 2:**
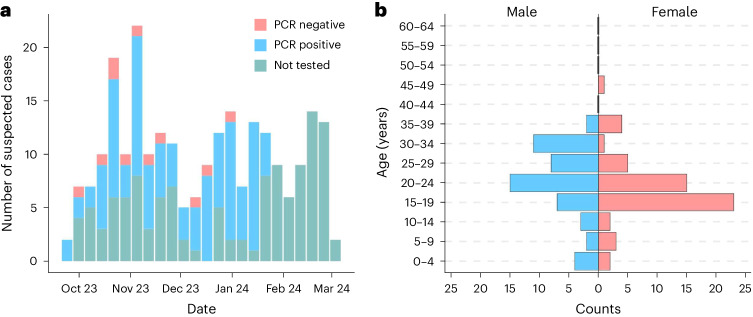
Epidemiologic curve and mpox cases disaggregated by age and sex, Kamituga, DRC. **a**, Epidemiologic curve of Kamituga mpox outbreak (October 2023 to March 2024), DRC. **b**, Counts of suspected mpox cases disaggregated by age and sex (male in blue, female in pink).

Near-full-length MPXV genome sequences, including 12 with genome coverage above 99%, 3 between 95% and 99%, 2 between 90% and 95% and 5 between 80% and 89% were obtained from 22 patients examined during the outbreak investigation. Genomic analysis revealed that all MPXV strains isolated from Kamituga clustered tightly with each other on a distinct lineage from previously sequenced clade I MPXV (Fig. 2a). The new lineage increases the known diversity of clade I by an additional 54%. Within the clade that previously encompassed the entirety of clade I, the maximum pairwise patristic distance totaled 4.89 × 10^−4^ substitutions per site (equivalent to ~96 individual nucleotide changes). With the inclusion of genomes from the Kamituga outbreak, this increased to 7.55 × 10^−4^ substitutions per site, or ~149 nucleotide changes. Sequences from two MPXV samples, collected in 2011 and 2012 during an mpox outbreak in North Kivu and South Kivu provinces^15^, clustered with the genomes sequenced in this study. However, due to limited genome coverage, their precise phylogenetic position relative to the Kamituga cluster could not be reliably determined.

The 22 MPXV genome sequences from Kamituga formed a compact cluster with 9 reconstructed mutations (Fig. 2b,c). Five of these mutations were consistent with APOBEC3-mediated cytosine deamination, indicating human-to-human transmission. Given the 8% rate of APOBEC3-like mutations in clade I MPXV in reservoir hosts, fewer than one APOBEC3-type mutation would be expected out of nine^7^. Furthermore, if MPXV in this cluster continued to evolve as observed in rodent host species, the binomial probability of observing five or more APOBEC3 mutations would be 0.0003. The predominance of APOBEC3-type mutations supports the finding that this entire cluster resulted from human-to-human transmission, while the low genomic diversity indicates a recent outbreak.

Molecular clock analysis, using the rate of evolution estimated for the human clade IIb MPXV outbreak^7^, estimates that the most recent common ancestor of the Kamituga genomes existed around mid-September 2023, consistent with the earliest reported cases. However, the credible intervals of this estimate cannot rule out a date as early as July 2023 (mean estimate 13 September 2023 with 95% highest posterior density intervals of 9 July 2023 to 3 October 2023). The estimated exponential growth rate was 10.8 per year (doubling time 23 days), but the 95% highest posterior density intervals include zero.

To compare strains isolated in Kamituga with those from the rest of the country, we included an additional 25 isolates from other provinces, collected from late 2023 to early 2024. These sequences aligned with previous clade I diversity (proposed to be identified as clade Ia), indicating no connection to the Kamituga mpox outbreak (Fig. 2b). Furthermore, the proportion of APOBEC3-type mutations compared to other mutations in samples collected outside the Kamituga area was only 15.3% (15 of 98 observed mutations), suggesting limited human-to-human transmission in those areas.

This report describes a novel clade I MPXV lineage linked to sustained human-to-human transmission in an ongoing outbreak in eastern DRC. Identification of APOBEC3-related mutations—a hallmark of MPXV human-to-human transmission^7^—bolsters this assertion. Due to its distinct geographical location and phylogenetic divergence, we propose naming this lineage clade Ib, with the previously described clade I renamed clade Ia (clade I now encompassing both Ia and Ib). The distinction between clades IIa and IIb was made on the basis of a similar rationale^16^.

Of note, a large ~1 kbp deletion in the MPXV genome (Δ19,128–20,270 coordinates relative to the clade I reference genome, GenBank accession NC_003310) has been reported in genome sequences from Kamituga linked to this outbreak^13^, specifically in the OPG032 gene and interfering with the clade I-specific diagnostic PCR originally developed by Li et al.^17^ The new genomes associated with the Kamituga outbreak also contain this deletion, implying that all genomes sampled from proposed clade Ib thus far exhibit this deletion and will not amplify with the specific diagnostic. Conversely, the MPXV clade Ia genomes we sampled from 2023/2024 did not contain this deletion. Two mpox samples from a 2011–2012 outbreak in North Kivu and South Kivu provinces^15^ clustered with the Kamituga outbreak genomes, but only five gene fragments were sequenced (with no coverage of the deleted region). We cannot therefore affirm whether this is a recent deletion specific to this outbreak or whether it is a viral feature of a nonhuman animal reservoir for this lineage. Although the proposed clade Ib is currently only represented by genomes from the Kamituga outbreak, the short fragmentary sequences isolated from earlier zoonotic mpox cases in the area suggest that this lineage probably preexisted in a local, nonhuman animal reservoir^15^.

Strengths of our study include novel and high-quality viral genomic sequencing performed at quality-controlled national and international laboratories in endemic mpox regions. However, limitations include the sparse availability of comprehensive clinical and demographic patient information in this retrospective case series. Despite these constraints, our data suggest that transmission in this outbreak was primarily linked to sexual contact: first, most affected individuals were adolescents and young adults, unlike previous DRC mpox outbreaks, in which children <15 years were most affected^18,19^; second, professional sex workers were disproportionately affected; finally, hospital records indicate that most cases presented with genital lesions compatible with viral infection.

The sustained spread of clade I MPXV in Kamituga, a densely populated, poor mining region, raises important concerns. The local healthcare infrastructure is ill-equipped to handle a large-scale epidemic, compounded by limited access to external aid. The 241 reported cases are probably an underestimate of the true incidence of mpox cases occurring in the area. In conversations with local healthcare workers, they reported that many additional people in the community had mpox symptoms but did not seek care.

Frequent travel occurs between Kamituga and the nearby city of Bukavu, with subsequent movement to neighboring countries such as Rwanda and Burundi. Moreover, sex workers operating in Kamituga represent several nationalities and frequently return to their countries of origin. Although there is no current evidence of wider dissemination of the outbreak, the highly mobile nature of this mining population poses a substantial risk of escalation beyond the current area and across borders. The international spread of clade I MPXV is particularly concerning due to its higher virulence compared to clade II. Among the 241 cases documented, two patients died, indicating a higher case fatality rate than the global clade IIb outbreak but lower than the current clade Ia outbreaks (4.6% reported among suspect cases in the DRC in 2024). This may be because clade Ib more often affects adults compared to clade Ia. However, additional data are needed to accurately assess clade Ib infection severity.

Our data confirm human-to-human transmission in MPXV genomes from Kamituga, supporting epidemiological findings of sexual contact transmission. However, this cluster of patients in Kamituga represents a minority of mpox cases reported during the 2022/2023 MPXV surge in the DRC. Genomic evidence demonstrates that most cases elsewhere in the country were not due to a single human outbreak or increased transmissibility from virus changes but instead due to several independent spillover events with subsequent secondary and onward transmission from reservoir hosts. The reason for this nationwide surge in cases remains unclear, and further investigation is needed to understand the drivers of these spillover events.

The situation in Kamituga mirrors the 2017–2018 outbreak of clade IIb MPXV in Nigeria. Without intervention, this localized outbreak has the potential to spread both nationally and internationally. Urgent measures are needed, including intensified local surveillance, enhanced community engagement and case management, and targeted mpox vaccination for individuals at risk, such as contacts of index cases, healthcare workers and key populations, including sex workers and men who have sex with men^20^.

Given the recent history of Mpox outbreaks in the DRC, urgent action is required from both endemic countries and the international community to prevent the broader geographic spread of clade I MPXV, both regionally and globally, with particular emphasis on addressing the needs of vulnerable key populations. Consequently, we have established the African-led, multidisciplinary, multicountry Mpox Research Consortium (MpoxReC) with the overarching goal of establishing a research network to advance the elimination of Mpox as a public health concern^20^. Key research priorities include further characterization of the new MPXV clade Ib, understanding transmission modes and disease severity, and evaluating point-of-care rapid diagnostic tests, prevention and treatment strategies accessible to populations at risk of severe disease. This comprehensive approach aims to improve both individual and epidemic outcomes.

## Methods

### Research ethics committee approval

This study used anonymized data and samples from mpox surveillance activities, outbreak response surveys and retrospective medical chart reviews. The secondary use of surveillance data and samples was approved by the Ethics Research Committee of the University of Kinshasa School of Public Health (approval ID ESP/CE/78/2024). As part of surveillance activities, individuals were investigated, and diagnostic samples were collected by health officials from the national mpox program or provincial health authorities. Patients consented orally to the collection of information and samples. Since the analyses conducted in this study were done retrospectively and the information and diagnostic samples were collected for surveillance and clinical care purposes, no written informed consent for research was provided by the patients.

### Population and setting

This study included patients hospitalized at the General Hospital of Kamituga, a gold mining town with approximately 242,000 inhabitants.

### Sampling and data collection

In January 2024, a multidisciplinary team comprising representatives from the Ministry of Public Health, provincial health authorities, the National Institute for Biomedical Research (INRB) and the Institute of Tropical Medicine investigated the mpox outbreak in Kamituga. We collated mpox monitoring data collected by provincial surveillance authorities. Local surveillance teams investigated and collected data from each suspected mpox case using a standardized Ministry of Public Health and Prevention form, which contains questions on demographics (age, sex, residence, profession and nationality), clinical symptoms, outcome and type of sample and collection date. For additional clinical data, we reviewed medical records of patients with suspected mpox admitted to the hospital between 6 October 2023 and 23 January 2024.

We then conducted interviews with local HCWs and local provincial and municipal health authorities. The INRB team interviewed and examined patients with suspected mpox, collecting samples from blood, skin lesions and oropharyngeal swabs for molecular diagnosis. Real-time PCR assays were performed at INRB Goma using the Light Mix Modular Monkeypox virus kit (Roche) according to the manufacturer’s instructions.

The following criteria were used for defining suspected mpox cases: an individual with a vesicular or pustular rash with deep-seated, firm pustules and ≥1 of the following symptoms: fever preceding the eruption, lymphadenopathy (inguinal, axillary or cervical) or pustules or crusts on hand palms or foot soles. A confirmed mpox case was identified when laboratory testing confirmed the presence of MPXV in the patient’s specimen, by real-time PCR and/or sequencing, following the diagnosis of a suspected case^21^. However, not all suspected cases of mpox were confirmed through PCR testing owing to resource constraint in this setting.

### MPXV genome sequencing

Sequencing was performed at the INRB’s Pathogen Genomics lab in Kinshasa. Samples testing positive with cycle threshold values <31 underwent sequencing using the Illumina DNA Prep with Enrichment kit (Illumina) and Comprehensive Viral Research Panel (Twist Biosciences). Following the manufacturer’s protocol, libraries were prepared and loaded onto the NextSeq 2000 sequencer. FASTQ files were processed through GeVarLi (https://forge.ird.fr/transvihmi/nfernandez/GeVarLi), CZid (https://czid.org/) and iVar pipelines for read quality control, consensus genome coverage and variant calling using a clade I reference genome (an early MPXV genome from DRC, accession NC_003310).

### Phylogenetic analysis

We compiled a dataset including all high-quality clade I MPXV genome sequences from GenBank (accessions and author list in Supplementary Table 2) and used clade IIa MPXV genomes from Nigeria dated 1978 and 1971 as outgroups (accession numbers KJ642615 and KJ642617, respectively). We estimated a maximum likelihood phylogeny using IQ-TREE 2 version 2.2.5 (ref. ^22^) with the HKY substitution model (Supplementary Fig. 1)^23^. Ancestral reconstruction was performed for each internal node on phylogeny using IQ-TREE 2, enabling mapping of single nucleotide polymorphisms (SNPs) along branches. SNPs were categorized on the basis of whether they were consistent with the signature of APOBEC3 editing, assuming this process induced specific mutations (TC → TT and GA → AA). To estimate the date of the most recent common ancestor of the Kamituga MPXV genomes and, thus, a bound on when the outbreak started, we followed the method described by O’Toole et al.^7^ implemented in BEAST v1.10.4^24^ with BEAGLE v4.0^25^. This approach divides the alignment into a partition containing all the sites that could potentially be edited by APOBEC3 and a partition containing the remaining sites. This allows a faster rate of evolution for the accumulation of APOBEC3-induced mutations than the background rate resulting from replication errors. As there is insufficient temporal information in these data to independently estimate the rate of evolution, we constrained the rates of these two partitions using posterior estimates from O’Toole et al.^7^ (approximated by a normal with mean 1.3 × 10^−4^ substitutions per site per year and standard deviation 1.0 × 10^−4^; and mean 4.2 × 10^−6^ and standard deviation 0.49 × 10^−6^, respectively). We used an exponential growth coalescent model as a prior on the tree. Finally, we estimated a maximum likelihood phylogeny of a sample of all clades I, IIa and Ib to look for relative divergence within and between clades for comparison with the divergence of putative clades Ia and Ib (Supplementary Fig. 2).

### Reporting summary

Further information on research design is available in the Nature Portfolio Reporting Summary linked to this article.

## Online content

Any methods, additional references, Nature Portfolio reporting summaries, source data, extended data, supplementary information, acknowledgements, peer review information; details of author contributions and competing interests; and statements of data and code availability are available at 10.1038/s41591-024-03130-3.

## Supplementary information

Supplementary Information Supplementary Figs. 1 and 2 and Tables 1–4.

Reporting Summary

## Data Availability

All epidemiological data produced in this study are deidentified and available upon request to the authors. Sequencing data are publicly accessible via GitHub at https://github.com/inrb-labgenpath/Mpox_sequencing_Kamituga, and the 47 complete or near-full-length MPXV genomes from this study have been deposited in the NCBI GenBank repository under accession numbers PP601182–PP601228 (Supplementary Tables 3 and 4).
