# Supplementary material for: Sustained human outbreak of a new MPXV clade I lineage in eastern Democratic Republic of the Congo
Source: Nat Med. 2024 Jun 13;30(10):2791–5. doi: 10.1038/s41591-024-03130-3 (PMC11485229; doi:10.1038/s41591-024-03130-3)
Supplement: Supplementary file 1 — Supplementary Figs. 1 and 2 and Tables 1–4. [file 41591_2024_3130_MOESM1_ESM.pdf]

nature medicine

Supplementary information https://doi.org/10.1038/s41591-024-03130-3

Sustained human outbreak of a new MPXV clade I lineage in eastern Democratic Republic of the Congo

In the format provided by the authors and unedited

<u>Supplemental Table 1</u> | Clinical and demographic characteristics of mpox suspected and confirmed cases

Characteristic	Suspected cases n = 241	Confirmed cases n = 108
Health zone where the diagnosis was made – no. (%)		
Kamituga	224 (93%)	103 (95%)
Other	17 (7.1%)	5 (4.6%)
Age (years) – median (IQR)	21 (18, 28)	22 (18, 27)
Sex – no. (%)		
Female	128 (54%)	56 (52%)
Male	111 (46%)	52 (48%)
Unknown	2	0
Professional sex worker – no. (%)	71 (29.5%)	31 (28.7% %)
Smallpox vaccinated – no. (%)		
No	239 (100%)	108 (100%)
Unknown	2	0
Nationality – no. (%)		
Congolese	217 (99%)	92 (99%)
Rwandese	1 (0.5%)	1 (1.1%)
Burundese	2 (0.9%)	0 (0%)
Unknown	21	15
Hospitalized – no. (%)		
Yes	217 (91%)	104 (96%)
No	22 (9.2%)	4 (3.7%)
Unknown	2	
Bedridden – no. (%)		
Yes	9 (3.8%)	10 (9.3%)
No	224 (95%)	98 (91%)
Unknown	3	0
Fever at presentation – no. (%)		
Yes	148 (62%)	64 (59%)
No	91 (38%)	44 (41%)
Unknown	2	0 (0%)
Skin rash at presentation – no. (%)		
Yes	239 (100%)	108 (100%)
No	0 (0%)	0 (0%)
Unknown	0	0

Cough at presentation – no. (%)		
Yes	41 (18%)	20 (19%)
No	191 (82%)	85 (81%)
Unknown	9	3
Adenopathy at presentation – no. (%)		
Yes	99 (42%)	45 (42%)
No	138 (58%)	62 (58%)
Unknown	4	1
Throat ache at presentation – no. (%)		
Yes	57 (24%)	29 (27%)
No	180 (76%)	78 (73%)
Unknown	4	1
Oral ulcers at presentation – no. (%)		
Yes	24 (10%)	13 (12%)
No	213 (90%)	94 (88%)
Unknown	4	1
Headache at presentation – no. (%)		
Yes	43 (18%)	19 (18%)
No	194 (82%)	88 (82%)
Unknown	4	1
Myalgia at presentation – no. (%)		
Yes	41 (17%)	20 (19%)
No	196 (83%)	87 (81%)
Unknown	4	1
Fatigue at presentation – no. (%)		
Yes	30 (13%)	19 (18%)
No	207 (87%)	88 (82%)
Unknown	4	1
Conjunctivitis at presentation – no. (%)		
Yes	9 (3.8%)	8 (7.4%)
No	229 (96%)	100 (93%)
Unknown	3	0

Supplemental Table 2 | Previously published MPXV genomes used in this study with NCBI Genbank accession number (Accession), country of origin (Country), top level administration region (Admin 1), date of sample collection to highest known precision (Date), reference for the genome sequence (Genome Reference), and reference for case description were available (Case Reference).

Accession	Country	Admin 1	Date	Genome Reference	Case Reference
KJ642618	Cameroon	Centre	1989-12-11	Nakazawa et al. 2015	Tchokoteu et al. 1991
KJ642613	DRC	Equateur	1970-09-01	Nakazawa et al. 2015	Ladnyj et al. 1972
DQ011155	DRC	Sankuru	1978-11-09	Likos et al. 2005	Breman et al. 1980
NC_003310	DRC	Sankuru	1996	Senkevich et al. 2021	Mukinda et al. 1997
DQ011154	Congo	Likouala	2003-06-09	Likos et al. 2005	Likos et al. 2005
KJ642612	DRC	Mongala	1986	Nakazawa et al. 2015	Jezek et al. 1987
KC257460	DRC	Mongala	1985	Nakazawa et al. 2015	Jezek et al. 1987
KJ642619	Gabon	Moyen-Ogooue	1987-06	Nakazawa et al. 2015	Müller et al. 1988
JX878417	DRC	Sankuru	2006-12-14	Kugelman et al. 2014	Kugelman et al. 2014
JX878408	DRC	Sankuru	2006-10-31	Kugelman et al. 2014	Kugelman et al. 2014
JX878423	DRC	Sankuru	2007-03-25	Kugelman et al. 2014	Kugelman et al. 2014
JX878424	DRC	Sankuru	2007-03-25	Kugelman et al. 2014	Kugelman et al. 2014
JX878426	DRC	Sankuru	2007-05-27	Kugelman et al. 2014	Kugelman et al. 2014
JX878413	DRC	Sankuru	2006-12-14	Kugelman et al. 2014	Kugelman et al. 2014
JX878414	DRC	Sankuru	2006-12-14	Kugelman et al. 2014	Kugelman et al. 2014
JX878415	DRC	Sankuru	2006-12-26	Kugelman et al. 2014	Kugelman et al. 2014
JX878416	DRC	Sankuru	2006-12-26	Kugelman et al. 2014	Kugelman et al. 2014
JX878421	DRC	Sankuru	2007-03-22	Kugelman et al. 2014	Kugelman et al. 2014
JX878422	DRC	Sankuru	2007-03-20	Kugelman et al. 2014	Kugelman et al. 2014

JX878425	DRC	Sankuru	2007-04-20	Kugelman et al. 2014	Kugelman et al. 2014
JX878427	DRC	Sankuru	2007-05-25	Kugelman et al. 2014	Kugelman et al. 2014
JX878428	DRC	Sankuru	2007-06-30	Kugelman et al. 2014	Kugelman et al. 2014
JX878429	DRC	Sankuru	2007-09-04	Kugelman et al. 2014	Kugelman et al. 2014
JX878418	DRC	Sankuru	2007-01-02	Kugelman et al. 2014	Kugelman et al. 2014
JX878419	DRC	Sankuru	2007-12-10	Kugelman et al. 2014	Kugelman et al. 2014
JX878420	DRC	Sankuru	2007-02-26	Kugelman et al. 2014	Kugelman et al. 2014
JX878407	DRC	Sankuru	2006-10-09	Kugelman et al. 2014	Kugelman et al. 2014
JX878409	DRC	Sankuru	2006-11-09	Kugelman et al. 2014	Kugelman et al. 2014
JX878410	DRC	Sankuru	2006-11-24	Kugelman et al. 2014	Kugelman et al. 2014
JX878411	DRC	Sankuru	2006-11-30	Kugelman et al. 2014	Kugelman et al. 2014
JX878412	DRC	Sankuru	2006-11-30	Kugelman et al. 2014	Kugelman et al. 2014
MT724770	DRC	Tshopo	2014	Mariën et al. 2021	Mariën et al. 2021
MT724772	DRC	Tshopo	2014	Mariën et al. 2021	Mariën et al. 2021
KP849469	DRC	Tshuapa	2008	Nakazawa et al. 2015	
KP849471	DRC	Yambuku	1985-07	Nakazawa et al. 2015	Khodakevich et al. 1986

<u>Supplemental Table 3</u> | NCBI Genbank accession number (Accession) for sequences generated during this study

Accession	Isolate	Clade	Country	Province	Health zone	Collection date
PP601182	23MPX1740C	Ia	DRC	Sud-Ubangi	Tandala	November 2023
PP601183	23MPX1766C	Ia	DRC	Sud-Ubangi	Bokonzi	November 2023
PP601184	23MPX1769C	Ia	DRC	Sud-Ubangi	Bokonzi	November 2023
PP601185	23MPX1786C	Ia	DRC	Sud-Ubangi	Tandala	December 2023
PP601186	23MPX1793V	Ia	DRC	Sud-Ubangi	Bangabola	December 2023
PP601187	23MPX1806V	Ia	DRC	Kinshasa	Kintambo	December 2023
PP601188	24MPX0008V	Ia	DRC	Sud-Ubangi	Bangabola	December 2023
PP601189	24MPX0009C	Ia	DRC	Sud-Ubangi	Bulu	December 2023
PP601190	24MPX0012C	Ia	DRC	Sud-Ubangi	Bulu	December 2023
PP601191	24MPX0014C	Ia	DRC	Sud-Ubangi	Bulu	December 2023
PP601192	24MPX0018V	Ia	DRC	Tshopo	Yahuma	January 2024
PP601193	24MPX0024C	Ia	DRC	Tshopo	Yahuma	December 2023
PP601194	24MPX0025V	Ia	DRC	Tshopo	Yahuma	December 2023
PP601195	24MPX0026V	Ia	DRC	Tshopo	Yahuma	December 2023
PP601196	24MPX0037V	Ia	DRC	Sud-Ubangi	Bulu	January 2024
PP601197	24MPX0038V	Ia	DRC	Sud-Ubangi	Bulu	January 2024
PP601198	24MPX0041V	Ia	DRC	Sud-Ubangi	Bulu	January 2024
PP601199	24MPX0164V	Ia	DRC	Tshopo	Basoko	January 2024
PP601200	24MPX0166C	Ia	DRC	Tshopo	Basoko	January 2024
PP601201	24MPX0168V	Ia	DRC	Tshopo	Yakusu	January 2024
PP601202	24MPX0169C	Ia	DRC	Tshopo	Yakusu	January 2024
PP601203	24MPX0174C	Ia	DRC	Tshopo	Yakusu	January 2024
PP601204	24MPX0175V	Ia	DRC	Tshopo	Yakusu	January 2024
PP601205	24MPX0188V	Ia	DRC	Equateur	Lotumbe	January 2024
PP601206	24MPX0194V	Ia	DRC	Equateur	Wangata	January 2024
PP601207	24MPX0198V	Ib	DRC	Sud-Kivu	Kamituga	January 2024
PP601208	24MPX0201V	Ib	DRC	Sud-Kivu	Kamituga	January 2024
PP601209	24MPX0203V	Ib	DRC	Sud-Kivu	Kamituga	January 2024
PP601210	24MPX0205V	Ib	DRC	Sud-Kivu	Kamituga	January 2024
PP601211	24MPX0206V	Ib	DRC	Sud-Kivu	Kamituga	January 2024
PP601212	24MPX0207Or	Ib	DRC	Sud-Kivu	Kamituga	January 2024
PP601213	24MPX0209V	Ib	DRC	Sud-Kivu	Kamituga	January 2024

PP601214	24MPX0214V	Ib	DRC	Sud-Kivu	Kamituga	January 2024
PP601215	24MPX0217V	Ib	DRC	Sud-Kivu	Kamituga	January 2024
PP601216	24MPX0220V	Ib	DRC	Sud-Kivu	Kamituga	January 2024
PP601217	24MPX0221V	Ib	DRC	Sud-Kivu	Kamituga	January 2024
PP601218	24MPX0223C	Ib	DRC	Sud-Kivu	Kamituga	December 2023
PP601219	24MPX0224C	Ib	DRC	Sud-Kivu	Kamituga	December 2023
PP601220	24MPX0226V	Ib	DRC	Sud-Kivu	Kamituga	December 2023
PP601221	24MPX0228V	Ib	DRC	Sud-Kivu	Kamituga	December 2023
PP601222	24MPX0230C	Ib	DRC	Sud-Kivu	Kamituga	December 2023
PP601223	24MPX0231V	Ib	DRC	Sud-Kivu	Kamituga	December 2023
PP601224	24MPX0239V	Ib	DRC	Sud-Kivu	Kamituga	January 2024
PP601225	24MPX0240V	Ib	DRC	Sud-Kivu	Kamituga	January 2024
PP601226	24MPX0242V	Ib	DRC	Sud-Kivu	Kamituga	January 2024
PP601227	RDC-NKV-GOM-MPOX-004	Ib	DRC	Sud-Kivu	Kamituga	October 2023
PP601228	RDC-NKV-GOM-MPOX-010	Ib	DRC	Sud-Kivu	Kamituga	October 2023

<u>Supplemental Table 4</u> | Reconstructed Mutations in Outbreak Genoms

Genomes	Nucleotide	Gene	Amino Acid	APOBEC3
242V, 0207Or	C4025T			TC->TT
242V, 0198V, 0239V, 0220V, 0217V, 206V, 0207Or, 0205V	C13862T	OPG025	E528K	TC->TT
0240V	G57493A	MPXVgp059	S28L	GA->AA
0230C	G89161A			GA->AA
0240V, 0231V, 0230C, 0228V, 0224C, 0221V, 0209V, 0201V	G115644A			GA->AA
0230C	A127779C	OPG145	K464E	
All but October	C168528A			
0226V, 0223C	A170739G	OPG193	T249A	
0221V	G186202A			

1 Genomes that share this mutation
2 Nucleotide position in genome
3 Gene for non-synonymous mutations
4 Amino acid residue mutation
5 APOBEC3 context (not APOBEC3 if missing)

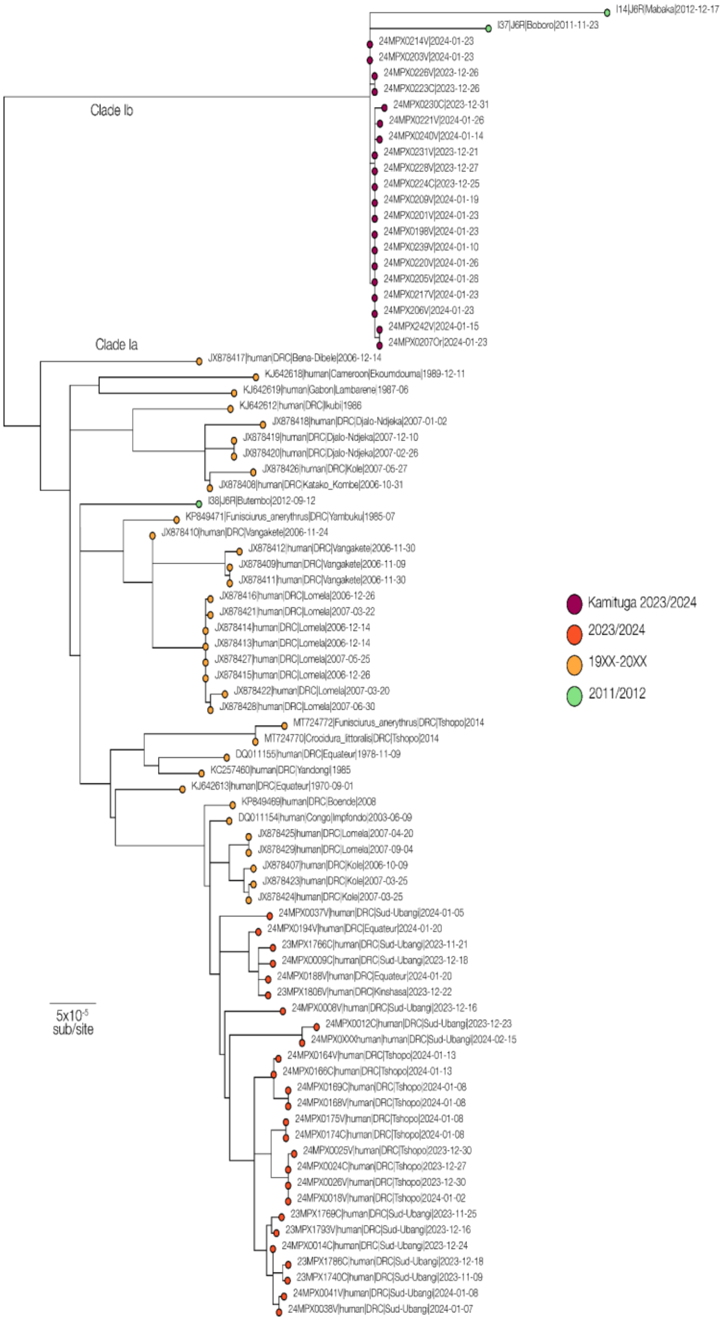

Supplemental Figure 1 | We estimated a labeled maximum likelihood phylogeny using IQTREE2, including all high quality MPXV Clade I genome sequences available on Genbank. We also include a set of sequences constructed from fragments sequenced from an outbreak in Sud-Kivu and Nord-Kivu, reported in 2011/2012 (McCollum et al 2015, doi: 10.4269/ajtmh.15-0095). These fragments represent only a small portion of the genome (five genes); the samples' exact positions on the phylogeny cannot be reliably

reconstructed. With the available data, however, we can assert that two of the samples cluster with the recent MPXV sequences from Sud-Kivu (proposed Clade Ib), and, although divergent from the current outbreak, they likely represent some of the reservoir diversity present in the area surrounding the Kivu provinces (I14 and I37). The third sequenced sample from the 2011/2012 outbreak (I38) does not cluster with this diversity and lies in the distinct Clade Ia.

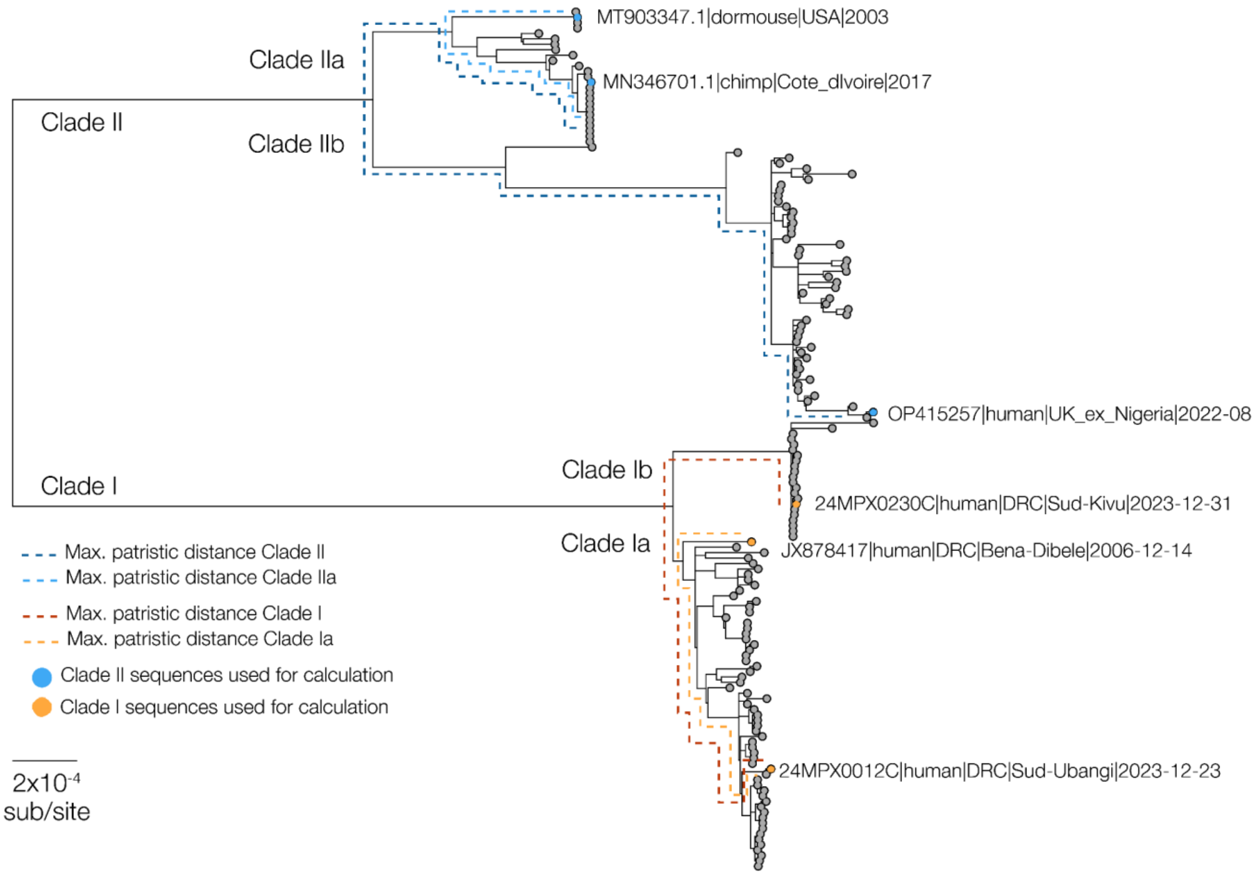

Supplemental Figure 2 | MPXV clades are indicated, including proposed Clade Ib, in the maximum likelihood phylogeny used to calculate maximum patristic distance and the increase in that with the addition of Clade IIb to Clade II and Clade IIb to Clade I. The sequences algorithmically identified for the calculation are indicated, and the backbone used for the pairwise calculations are marked with a dashed line.
