# Supplementary material for: Sustained human outbreak of a new MPXV clade I lineage in eastern Democratic Republic of the Congo
Source: Nat Med. 2024 Jun 13;30(10):2791–5. doi: 10.1038/s41591-024-03130-3 (PMC11485229; doi:10.1038/s41591-024-03130-3)
Supplement: Supplementary file 2 — Reporting Summary [file 41591_2024_3130_MOESM2_ESM.pdf]

nature portfolio

Corresponding author(s): Jean B. Nachega, MD, PhD, MPH, FRCP, FAAS

Last updated by author(s): Jun 5, 2024

Reporting Summary

Nature Portfolio wishes to improve the reproducibility of the work that we publish. This form provides structure for consistency and transparency in reporting. For further information on Nature Portfolio policies, see our Editorial Policies and the Editorial Policy Checklist.

Statistics

For all statistical analyses, confirm that the following items are present in the figure legend, table legend, main text, or Methods section.

n/a	Confirmed	
☐	☒	The exact sample size (*n*) for each experimental group/condition, given as a discrete number and unit of measurement
☒	☐	A statement on whether measurements were taken from distinct samples or whether the same sample was measured repeatedly
☒	☐	The statistical test(s) used AND whether they are one- or two-sided<br>*Only common tests should be described solely by name; describe more complex techniques in the Methods section.*
☒	☐	A description of all covariates tested
☒	☐	A description of any assumptions or corrections, such as tests of normality and adjustment for multiple comparisons
☐	☒	A full description of the statistical parameters including central tendency (e.g. means) or other basic estimates (e.g. regression coefficient) AND variation (e.g. standard deviation) or associated estimates of uncertainty (e.g. confidence intervals)
☒	☐	For null hypothesis testing, the test statistic (e.g. *F*, *t*, *r*) with confidence intervals, effect sizes, degrees of freedom and *P* value noted<br>*Give P values as exact values whenever suitable.*
☒	☐	For Bayesian analysis, information on the choice of priors and Markov chain Monte Carlo settings
☒	☐	For hierarchical and complex designs, identification of the appropriate level for tests and full reporting of outcomes
☒	☐	Estimates of effect sizes (e.g. Cohen's *d*, Pearson's *r*), indicating how they were calculated

Our web collection on statistics for biologists contains articles on many of the points above.

Software and code

Policy information about availability of computer code

Data collection

SAMPLING AND DATA COLLECTION
In January 2024, a multidisciplinary team comprising representatives from the Ministry of Public Health, provincial health authorities, the National Institute for Biomedical Research (INRB) and the Institute of Tropical Medicine (ITM) investigated the mpox outbreak in Kamituga. We collated mpox monitoring data collected by provincial surveillance authorities. Local surveillance teams investigated and collected data from each suspected mpox case using a standardized Ministry of Public Health and Prevention form, which contains questions on demographics (age, sex, residence, profession, nationality), clinical symptoms, outcome, and type of sample and collection date. For additional clinical data, we reviewed medical records of patients with suspected mpox admitted to the hospital between October 6, 2023, and January 23, 2024.

We then conducted interviews with local HCWs and local provincial and municipal health authorities. The INRB team interviewed and examined patients with suspected mpox, collecting samples from blood, skin lesions, and oropharyngeal swabs for molecular diagnosis. Real-time PCR assays were performed at INRB Goma using the Light Mix Modular Monkeypox virus kit (Roche, Germany) according to manufacturer’s instructions.

The DRC national case definition was used for defining suspected mpox cases: an individual with a vesicular or pustular rash with deep-seated, firm pustules, and ≥1 of the following symptoms: fever preceding the eruption, lymphadenopathy (inguinal, axillary, or cervical), or pustules or crusts on hand palms or foot soles. A confirmed mpox case was identified when laboratory testing confirmed the presence of MPXV in the patient's specimen, by real-time PCR and/or sequencing, following the diagnosis of a suspected case.21 However, not all suspected cases of mpox were confirmed through PCR testing due to resource constraint in this setting.

MPXV GENOME SEQUENCING

Sequencing was performed at the INRB's Pathogen Genomics lab in Kinshasa. Samples testing positive with Cycle threshold (Ct) values <31 underwent sequencing using Illumina DNA Prep with Enrichment kit (Illumina) and Comprehensive Viral Research Panel (Twist Biosciences). Following the manufacturer's protocol, libraries were prepared and loaded onto the NextSeq 2000 sequencer. FASTQ files were processed through GeVarLi (https://forge.ird.fr/transvihmi/nfernandez/GeVarLi), CZid (https://czid.org/) and iVar pipelines for read quality control, consensus genome coverage, and variant calling using a Clade I reference genome (an early MPXV genome from DRC, accession: NC_003310).

Data analysis

PHYLOGENETIC ANALYSIS
We compiled a dataset including all high-quality Clade I MPXV genome sequences from GenBank (accessions and author list in Extended Data Table 1) and used Clade IIa MPXV genomes from Nigeria dated 1978 and 1971 as outgroups (accession numbers KJ642615 and KJ642617, respectively). We estimated a maximum likelihood phylogeny using IQ-TREE 2 version 2.2.522 with the HKY substitution model.23 Ancestral reconstruction was performed for each internal node on phylogeny using IQ-TREE 2, enabling mapping of single nucleotide polymorphisms (SNPs) along branches. SNPs were categorized based on whether they were consistent with the signature of APOBEC3-editing, assuming this process induced specific mutations (TC→TT and GA→AA). To estimate the date of the most recent common ancestor of the Kamituga MPXV genomes, and thus a bound on when the outbreak started, we followed the method described by O'Toole et al.7 implemented in BEAST v1.10.424 with BEAGLE v4.025. This approach divides the alignment into a partition containing all the sites that could potentially be edited by APOBEC3 and a partition containing the remaining sites. This allows faster rate of evolution for the accumulation of APOBEC3 induced mutations than the background rate resulting from replication errors. As there is insufficient temporal information in these data to independently estimate the rate of evolution, we constrained the rates of these two partitions using posterior estimates from O'Toole et al.7 (approximated by a normal with mean 1.3 × 10-4 substitutions/site/year and standard deviation 1.0 × 10-4; and mean 4.2 × 10-6 and standard deviation 0.49 × 10-6, respectively). We used an exponential growth coalescent model as a prior on the tree. Finally, we estimated a maximum likelihood phylogeny of a sample of all Clade I, IIa and Ib to look for relative divergence within and between clades for comparison with the divergence of putative Clades Ia and Ib (Extended Data Figure 2).

For manuscripts utilizing custom algorithms or software that are central to the research but not yet described in published literature, software must be made available to editors and reviewers. We strongly encourage code deposition in a community repository (e.g. GitHub). See the Nature Portfolio guidelines for submitting code & software for further information.

Data

Policy information about availability of data

All manuscripts must include a data availability statement. This statement should provide the following information, where applicable:

- Accession codes, unique identifiers, or web links for publicly available datasets
- A description of any restrictions on data availability
- For clinical datasets or third party data, please ensure that the statement adheres to our policy

DATA AVAILABILITY
All epidemiological data produced in this study are de-identified and available upon request to the authors. Sequencing data are publicly accessible at https://github.com/inrb-labgenpath/Mpox_sequencing_Kamituga, and the 47 complete or near full-length MPXV genomes from this study have been deposited in the NCBI GenBank repository under accession numbers PP601182-PP601228 (see Supplemental Table 2).

Human research participants

Policy information about studies involving human research participants and Sex and Gender in Research.

Reporting on sex and gender

The median age was 22 years (IQR 18-27) (Figure 1B). Children <15 years constituted 14.8% (16/108) of confirmed cases, individuals aged 15-30 years accounted for 67% (73/108), and 17.6% (19/108) of cases were individuals 30-49 years

Population characteristics

Among individuals with PCR-confirmed mpox, the majority were female (56/108, 51.9%) and the median age was 22 years (IQR 18-27) (Figure 1B). Children <15 years constituted 14.8% (16/108) of confirmed cases, individuals aged 15-30 years accounted for 67% (73/108), and 17.6% (19/108) of cases were individuals 30-49 years

Recruitment

Between September 29, 2023, and February 29, 2024, South Kivu's provincial surveillance authorities recorded 241 suspected cases that met the national case definition for mpox (Figure 1A). Most cases (93%) were in Kamituga Health Zone. Laboratory specimens from 119 (48.9%) of the patients hospitalized for mpox were collected for PCR testing. Of these, 108 (90.8%) were confirmed MPXV-positive. Demographic characteristics and clinical manifestations were similar between suspected and confirmed cases (Supplemental Table 1).

Ethics oversight

RESEARCH ETHICS COMMITTEE APPROVAL This study used anonymized data and samples from mpox surveillance activities, outbreak response surveys, and retrospective medical chart reviews. The secondary use of surveillance data and samples was approved by the Ethics Research Committee of the University of Kinshasa School of Public Health (Approval ID: ESP/CE/78/2024). As part of surveillance activities, individuals were investigated, and diagnostic samples were collected by. health officials from the national mpox program or provincial health authorities. Patients consented orally to the collection of information and samples. Since the analyses conducted in this study were done retrospectively and the information and diagnostic samples were collected for surveillance and clinical care purposes, no written informed consent for research was provided by the patients.

Note that full information on the approval of the study protocol must also be provided in the manuscript.

Field-specific reporting

Please select the one below that is the best fit for your research. If you are not sure, read the appropriate sections before making your selection.

☒ Life sciences ☐ Behavioural & social sciences ☐ Ecological, evolutionary & environmental sciences

For a reference copy of the document with all sections, see nature.com/documents/nr-reporting-summary-flat.pdf

Life sciences study design

All studies must disclose on these points even when the disclosure is negative.

Sample size	We performed a convenience sampling. This method involves taking available participants who meet the criteria rather than using a pre-determined sample size or a random sampling method. Convenience sampling is often used when researchers rely on readily available subjects and useful in preliminary research or situations with limited resources. The post hoc power analysis indicates a power of approximately 0.955, or 95.5%. This means that the study, with 108 confirmed MPXV-positive cases and assuming a medium effect size has a high probability (95.5%) of detecting a statistically significant effect if one exists.
Data exclusions	No data were excluded
Replication	Between September 29, 2023, and February 29, 2024, South Kivu's provincial surveillance authorities recorded 241 suspected cases of mpox. Of these, laboratory specimens were collected from 119 hospitalized patients for PCR testing. The PCR testing confirmed 108 cases as MPXV-positive. The study aimed to ensure reproducibility by standardizing the PCR testing protocol and consistently applying the national case definition for mpox across all recorded cases. Demographic characteristics and clinical manifestations were systematically recorded and analyzed to confirm consistency between suspected and confirmed cases. Each patient's sample was tested once, and no technical replicates were performed for the PCR testing.
Randomization	Not applicable as our study was not a clinical trial
Blinding	Not applicable as our study was not a clinical trial

Reporting for specific materials, systems and methods

We require information from authors about some types of materials, experimental systems and methods used in many studies. Here, indicate whether each material, system or method listed is relevant to your study. If you are not sure if a list item applies to your research, read the appropriate section before selecting a response.

Materials & experimental systems

n/a	Involved in the study	
☒	☐	Antibodies
☒	☐	Eukaryotic cell lines
☒	☐	Palaeontology and archaeology
☒	☐	Animals and other organisms
☐	☒	Clinical data
☒	☐	Dual use research of concern

Methods

n/a	Involved in the study	
☒	☐	ChIP-seq
☒	☐	Flow cytometry
☒	☐	MRI-based neuroimaging

Clinical data

Policy information about clinical studies

All manuscripts should comply with the ICMJE guidelines for publication of clinical research and a completed CONSORT checklist must be included with all submissions.

Clinical trial registration	Not applicable as our study was not a clinical trial
Study protocol	Study protocol can be obtained by sending an e-mail request to either of the corresponding authors: Professor Placide Mbala (mbalaplacide@gmail.com) or Professor Jean Nachega (jbn16@pitt.edu)
Data collection	SAMPLING AND DATA COLLECTION<br>In January 2024, a multidisciplinary team comprising representatives from the Ministry of Public Health, provincial health authorities, the National Institute for Biomedical Research (INRB) and the Institute of Tropical Medicine (ITM) investigated the mpox outbreak in Kamituga. We collated mpox monitoring data collected by provincial surveillance authorities. Local surveillance teams investigated and collected data from each suspected mpox case using a standardized Ministry of Public Health and Prevention form, which contains questions on demographics (age, sex, residence, profession, nationality), clinical symptoms, outcome, and type of sample and collection date. For additional clinical data, we reviewed medical records of patients with suspected mpox admitted to the hospital between October 6, 2023, and January 23, 2024.

We then conducted interviews with local HCWs and local provincial and municipal health authorities. The INRB team interviewed and examined patients with suspected mpox, collecting samples from blood, skin lesions, and oropharyngeal swabs for molecular diagnosis. Real-time PCR assays were performed at INRB Goma using the Light Mix Modular Monkeypox virus kit (Roche, Germany) according to manufacturer's instructions.
The DRC national case definition was used for defining suspected mpox cases: an individual with a vesicular or pustular rash with deep-seated, firm pustules, and ≥1 of the following symptoms: fever preceding the eruption, lymphadenopathy (inguinal, axillary, or cervical), or pustules or crusts on hand palms or foot soles. A confirmed mpox case was identified when laboratory testing confirmed the presence of MPXV in the patient's specimen, by real-time PCR and/or sequencing, following the diagnosis of a suspected case.21 However, not all suspected cases of mpox were confirmed through PCR testing due to resource constraint in this setting.

Outcomes

Primary outcome was participants with confirmed mpox via MPXV-PCR+. The DRC national case definition was used for defining suspected mpox cases: an individual with a vesicular or pustular rash with deep-seated, firm pustules, and ≥1 of the following symptoms: fever preceding the eruption, lymphadenopathy (inguinal, axillary, or cervical), or pustules or crusts on hand palms or foot soles. A confirmed mpox case was identified when laboratory testing confirmed the presence of MPXV in the patient's specimen, by real-time PCR and/or sequencing, following the diagnosis of a suspected case.21 However, not all suspected cases of mpox were confirmed through PCR testing due to resource constraint in this setting.
